# mirtronDB 2.0: enhanced database with novel mirtron discoveries

**DOI:** 10.1093/bioinformatics/btag114

**Published:** 2026-03-11

**Authors:** Fabiana Rodrigues de Goes, Matheus Fujimura Soares, Vitor Gregorio, Bruno Thiago de Lima Nichio, Alisson Gaspar Chiquitto, Flavia Lombardi Lopes, Mark Basham, Douglas Silva Domingues, Alexandre Rossi Paschoal

**Affiliations:** Rosalind Franklin Institute, Harwell Science and Innovation Campus, Didcot, OX11 0QS, United Kingdom; School of Veterinary Medicine, São Paulo State University (UNESP), Araçatuba, 16050-680, Brazil; Department of Computer Science, Federal University of Technology-Parana, Cornelio Procopio, 86300-000, Brazil; Department of Computer Science, Federal University of Technology-Parana, Cornelio Procopio, 86300-000, Brazil; Department of Computer Science, Federal University of Technology-Parana, Cornelio Procopio, 86300-000, Brazil; Federal Institute of Education, Science and Technology of Mato Grosso do Sul, Navirai, 79947-334, Brazil; School of Veterinary Medicine, São Paulo State University (UNESP), Araçatuba, 16050-680, Brazil; Rosalind Franklin Institute, Harwell Science and Innovation Campus, Didcot, OX11 0QS, United Kingdom; Department of Genetics, “Luiz de Queiroz” College of Agriculture, University of São Paulo, Piracicaba, São Paulo, 13418-900, Brazil; Rosalind Franklin Institute, Harwell Science and Innovation Campus, Didcot, OX11 0QS, United Kingdom; Department of Computer Science, Federal University of Technology-Parana, Cornelio Procopio, 86300-000, Brazil

## Abstract

**Motivation:**

MirtronDB provides a comprehensive and up-to-date resource for advancing mirtron research within RNA biology. Therefore, maintaining a specialized and continuously updated resource for mirtrons is essential to support ongoing discoveries and to serve as a key reference for researchers investigating the roles of mirtrons.

**Results:**

Here, we present mirtronDB 2.0, an enhanced version that expands both content and functionality. This version integrates mirtron data published between 2017 and 2025, increasing the number of documented mirtrons across various species. In addition, it incorporates newly predicted mirtrons identified through a robust pipeline that combines advanced bioinformatics and machine learning approaches, with specific coverage of six mammalian species. We have introduced new website features, including an interactive dashboard to enhance usability and facilitate intuitive data exploration. These rigorous updates consolidate mirtronDB as a key resource for mirtron to the RNA biology community.

**Availability and implementation:**

mirtronDB can be found under http://mirtrondb.cp.utfpr.edu.br/. The complete content of Database 2.0 and the source code for the analyses are also freely available in the FigShare repository: https://figshare.com/articles/dataset/MirtronDB_version2/29344775.

## 1 Introduction

Mirtrons are a subclass of pre-miRNAs encoded by a non-canonical pathway independent of the Drosha enzyme. Despite the existence of microRNA (miRNA) databases (de Amorim *et al.* 2022), e.g. miRBase (Kozomara *et al.* 2019) and MirGeneDB (Clarke *et al.* 2025), no resource was specifically dedicated to mirtrons. The available data were dispersed across publications, lacking standardization and centralization. To address this, the first version of mirtronDB (da Fonseca *et al.* 2019) introduced the only peer-reviewed and publicly accessible database dedicated to mirtrons, providing curated, species-specific data with standardized annotations. This highlights the importance of a continuously updated resource, prompting the development of this significantly expanded version.

In this work, we present mirtronDB 2.0, an updated resource featuring new content and enhanced functionality. Our systematic approach involved a comprehensive retrieval and integration of mirtron data from the recent literature, a rigorous curation process, and an *in silico* pipeline, based on similarity search and machine learning, to improve cross-species coverage and standardized annotation. We also introduce new website features, including an interactive dashboard, to improve usability and data exploration for the RNA biology community. To maintain consistency and standardization across the database while integrating newly collected mirtron data, we conducted a structured curation effort to review and remap the genomic coordinates of previously available and newly identified mirtrons to the latest Ensembl (Dyer *et al.* 2025) genome assemblies. To complement the literature-curated entries and explore the potential of discovering new mirtrons, we implemented an *in silico* prediction strategy across six mammalian species, identifying 165 potential novel homolog mirtrons. Finally, all data is open access from the download page on the website.

## 2 Materials and methods

**Figure 1:**
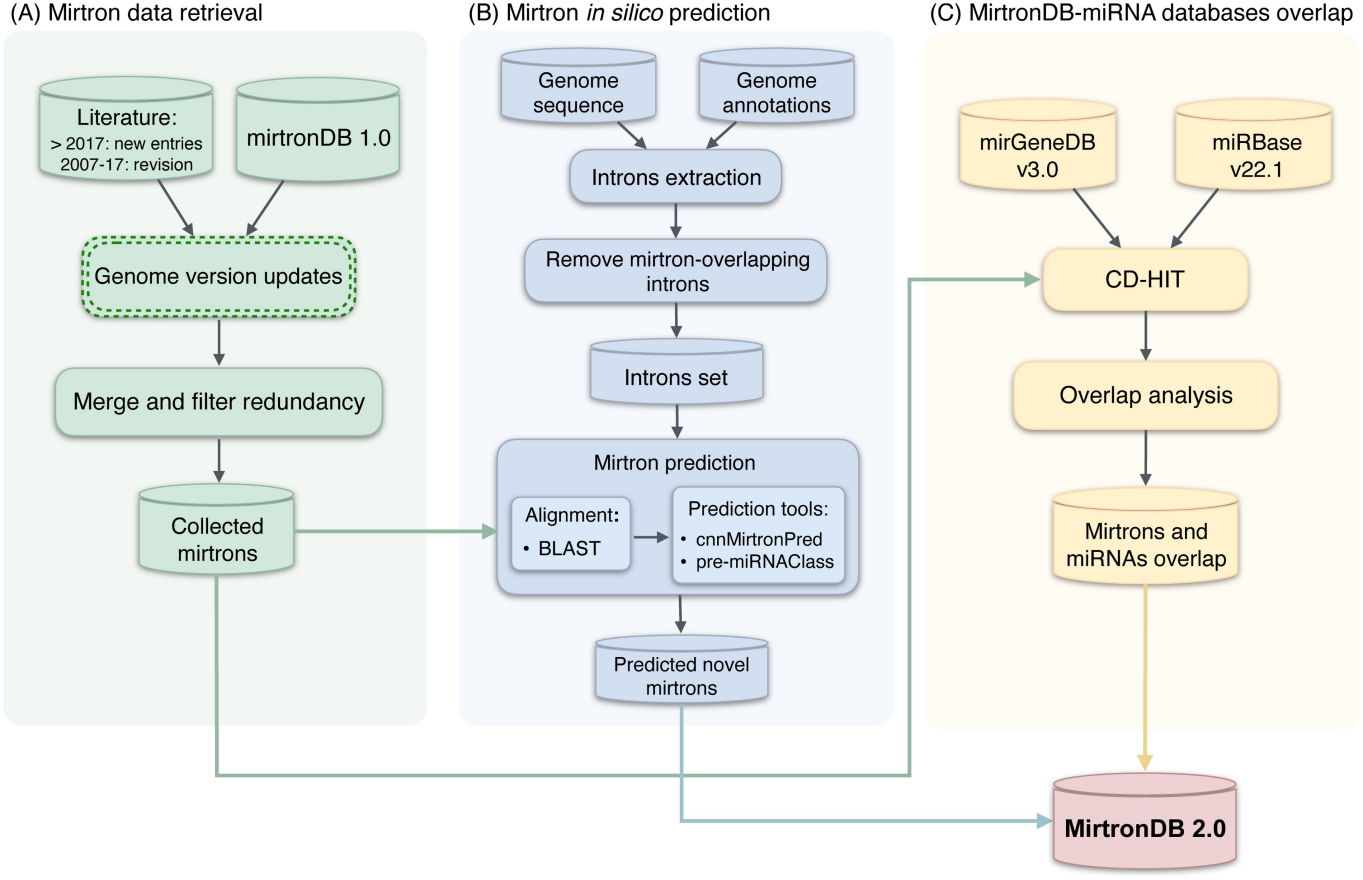
Overview of the mirtronDB 2.0 construction pipeline, comprising three main stages: (A) mirtron data retrieval, including review and update of mirtronDB 1.0 entries and collection of additional mirtrons reported in recent literature; (B) *in silico* prediction of mirtrons using similarity-search and machine learning tools to improve cross-species coverage and standardized annotation; and (C) comparison of retrieved mirtrons from (A) with reference miRNA databases to identify overlapping and unique entries.

The mirtronDB 2.0 pipeline integrates multiple data sources and computational strategies to ensure a comprehensive and high-quality dataset (Fig. 1). The process started with the review and update of mirtronDB 1.0 entries, along with the collection of additional mirtrons reported in the literature in recent years (Fig. 1A). Next, an *in silico* prediction pipeline was applied to identify similar mirtrons using bioinformatic and machine learning tools (Fig. 1B). Finally, we compared the entries from mirtronDB updated from literature only (Fig. 1A) with established miRNA databases to identify overlapping and unique entries (Fig. 1C). This structured approach ensures both the reliability and novelty of the database.

### 2.1 Mirtron data retrieval

We revisited studies published between 2007 and 2017 to review and recover data that had occasionally been missed in the mirtronDB 1.0 analyses (Table S1, available as supplementary data at *Bioinformatics* online, column “Revision”). In the end, we collected data from one article (Schamberger *et al.* 2012).

Next, we collected available mirtron data from articles published between 2017 and April 2025 by searching the National Center for the Biotechnology Information (NCBI) PubMed database using the query “(mirtron) AND (mirtrons)” with only the English language (Fig. 1A and Fig. S1, available as supplementary data at *Bioinformatics* online). This search yielded 42 articles (Collins *et al.* 2017, Monteleone and Lutz 2017, Baghdadi *et al.* 2018, Campbell *et al.* 2018, Hansen 2018, Ju *et al.* 2018, Mohammed *et al.* 2018, Rorbach *et al.* 2018, Titov and Vorozheykin 2018, Urbanek-Trzeciak *et al.* 2018, Zia and Flynt 2018, Bell *et al.* 2019, Cheng *et al.* 2019, da Fonseca *et al.* 2019, Kroupova *et al.* 2019, Zheng *et al.* 2019, Amourda and Saunders 2020, Dexheimer *et al.* 2020, Kim *et al.* 2020, Rivera *et al.* 2020, Yao *et al.* 2020, Zhao *et al.* 2020a, 2020b, Divisato *et al.* 2021, Mirzaei *et al.* 2021, Mohanta and Chakrabarti 2021, Orlans *et al.* 2021, Schamberger *et al.* 2021, Tasdelen and Sen 2021, Wong and Rasko 2021, de Amorim *et al.* 2022, Salim *et al.* 2022, Zapletal *et al.* 2022, Lee *et al.* 2023a, 2023b, Amaro *et al.* 2024, Buccheri *et al.* 2024, Gál *et al.* 2024, Khanal *et al.* 2024, Yao *et al.* 2024, Gao *et al.* 2025, Salim *et al.* 2025) (Table S1, available as supplementary data at *Bioinformatics* online). Next, we manually reviewed the full texts, supplementary materials, and references of all these articles to identify studies reporting novel mirtrons, selecting nine articles (Collins *et al.* 2017, Monteleone and Lutz 2017, Ju *et al.* 2018, Bell *et al.* 2019, Yao *et al.* 2020, Zhao *et al.* 2020a, Mirzaei *et al.* 2021, Amaro *et al.* 2024, Gao *et al.* 2025). For each selected study, we extracted information such as species, genomic coordinates, and genome sequences, including data presented exclusively in supplementary files. Finally, we removed all entries already present in mirtronDB 1.0 by cross-checking species information, genomic coordinates, and sequence data through manual curation (Fig. S1, available as supplementary data at *Bioinformatics* online).

### 2.2 mirtronDB updates

The mirtrons included in mirtronDB 1.0 and the newly identified mirtrons were subjected to an update process (Fig. 1A) to their genomic coordinates across all 33 species in the mirtronDB 2.0 (Table S2, available as supplementary data at *Bioinformatics* online). First, we assessed whether the genome assembly of each species was updated according to the most recent version available in the Ensembl database. For cases where the assembly was outdated, we followed this workflow (Fig. S2 and Document S1, available as supplementary data at *Bioinformatics* online):

1. The genomic coordinates were updated according to the most recent genome assembly available in Ensembl.
2. If the mirtron sequence was 100% similar in the previous genome assembly and at least 90% similar in the updated assembly, we chose to update the sequence. The 90% similarity threshold was chosen to tolerate assembly-related variation while still preserving the integrity of mirtron annotation.
3. If these criteria were not satisfied, we opted to use the second most updated genome assembly.

In addition to the described workflow, the presence of paralogs was also identified (Document S1, available as supplementary data at *Bioinformatics* online). When the same sequence was detected at two or more distinct genomic loci, it was classified as a paralog, and its name was updated accordingly (e.g. mirtron-1, mirtron-2) to reflect its multiple genomic occurrences.

### 2.3 Mirtron *in silico* prediction

To achieve a comprehensive prediction of potential cross-species coverage of similar mirtrons, we developed a computational pipeline (Fig. 1B) that begins with the extraction of intronic sequences based on genome assemblies and transcript annotations from the Ensembl database. For each transcript, exons were identified, and introns were defined as the regions between adjacent exons. To eliminate redundancy caused by transcript isoforms, duplicate introns were removed by comparing their genomic coordinates, strand, and chromosome. Intronic sequences were then retrieved using the *getfasta* command from Bedtools (Quinlan and Hall 2010) with default parameters. Finally, introns overlapping known mirtrons were excluded using Bedtools *intersect*, with strandedness enforced (parameter -s) and all other parameters set to default.

Next, sequence similarity searches were performed with BLAST (version 2.16.0+, NCBI), using the full mirtronDB 2.0 (all species) as the subject database and the extracted introns as queries. Alignments were filtered applying thresholds of at least 98% identity and coverage, where coverage was defined as the ratio between alignment length and subject length. To ensure consistency with known mirtron characteristics, candidates with intron lengths exceeding the maximum precursor length observed among known mirtrons (177 bp) were removed. These candidate introns filtered by length were then classified using two machine learning-based mirtron prediction tools: cnnMirtronPred Zheng *et al.* (2019) available at https://github.com/zhengxueming/cnnMirtronPred (commit ID: f2f22f0), and pre-miRNAClassification (Aziz and Hasan 2019) available at https://github.com/zahidemon/Pre-miRNA-Classification (commit ID: c02f743). Users could also download both tools from our FigShare (https://doi.org/10.6084/m9.figshare.29344775). The classification was performed using the pre-trained models available in their respective repositories, without any additional model training and using only the default execution parameters. The candidate introns were provided as input to the tools, which subsequently classified each sample as a mirtron or canonical miRNA. The selection of the two tools builds on previous work that analyzed the state of the art in mirtron classification (Gaspar Chiquitto *et al.* 2025). To account for distinct levels of prediction evidence, we adopted a confidence prediction scheme based on the agreement between the machine learning tools. Predictions supported by both tools (intersection) were classified as high confidence, whereas predictions identified by only one classifier were labeled as moderate confidence. This strategy allows a broader inclusion of candidate mirtrons while preserving interpretability of prediction reliability.

This procedure was carried out separately for each species. The analysis was conducted on six mammalian species: *B. taurus*, *C. familiaris*, *H. sapiens*, *M. musculus*, *M. mulatta*, and *P. troglodytes*.

### 2.4 Mirtrons and miRNAs similarity analysis

To assess the contribution of mirtronDB 2.0 in the context of existing miRNA annotations, we performed a comparative analysis between the entries from mirtronDB collected only from literature (Fig. 1A) and two widely used reference databases of annotated miRNAs: miRBase v22.1 (Kozomara *et al.* 2019) and MirGeneDB v3.0 (Clarke *et al.* 2025) (Fig. 1C). We used only mirtrons collected from literature because predicted mirtrons do not have mature information. This analysis served a dual purpose: (i) to quantify the extent of unique content in MirtronDB 2.0 by assessing how many mature mirtrons are not present in miRNA resources, thereby highlighting its novelty and (ii) to systematically annotate mirtrons that overlap with entries in these references, providing users with cross-references to their entries in other miRNA databases.

To identify overlapping sequences, pairwise sequence comparisons were performed using the CD-HIT-EST-2D tool (Huang *et al.* 2010), aligning mature mirtron sequences against miRBase and MirGeneDB entries. To ensure high-confidence matches, we applied a stringent threshold requiring a minimum of 9 nucleotides of overlap (parameter -n 9) and at least 98% sequence identity (parameter -c 0.98). Given the short length of mature miRNAs, the 98% identity ensures that only highly similar and potentially functionally related sequences are matched.

### 2.5 Target gene prediction

The human mirtron–mRNA interactions were predicted using the miRanda algorithm (Enright *et al.* 2003). Mature human mirtron sequences were retrieved from mirtronDB 2.0, and the 3’UTR sequences, used as the target dataset, were obtained from Ensembl BioMart (Kinsella *et al.* 2011).

The analysis was conducted with a minimum alignment score threshold of 140, a maximum free energy cutoff of −10 kcal/mol, and the strict seed pairing requirement to ensure high-confidence predictions.

## 3 Results

### 3.1 mirtronDB 2.0: database content

mirtronDB 2.0 presents a significant update from its previous version by incorporating new findings from the literature. Compared to version 1, the database expanded from 18 to 33 species and increased the number of mirtron entries from 1475 to 1589 (Table 1). These advances consolidate mirtronDB as a central resource for investigating mirtrons across species.

**Table 1:** Summary of content and functional improvements from the first (1.0) to the latest release (2.0) of mirtronDB.

	mirtronDB 1.0	mirtronDB 2.0
Articles	20	31
Species	18	33
Mirtrons entries	1475	1589
Mature mirtrons	2426	2470
Mirtron precursors	1407	1531
Predicted mirtrons	–	165
Cross-reference	miRBase	miRBase and MirGeneDB
Website	Non-interactive Statistics	Dashboard

### 3.2 Mirtron in silico prediction

The *in silico* prediction pipeline to improve cross-species coverage of similar mirtrons revealed substantial variability across species, both in the number of introns retained after alignment analysis and in the level of agreement between the classification tools (Table 2). Overall, the final number of predicted mirtrons was relatively low for most species, not only for high-confidence predictions (agreement between both tools), but also when considering moderate-confidence predictions. For example, in *H. sapiens*, 242 candidate introns passed the BLAST analysis; pre-miRNAClassification predicted up to 13 mirtrons, while cnnMirtronPred predicted 6. However, only 4 were identified with high confidence, highlighting the strictness of this agreement, and 2 with moderate confidence. In *M. mulatta*, the classifiers showed strong agreement, with 18 high-confidence mirtrons and 2 moderate-confidence predictions. A similar pattern was observed in *P. troglodytes*, which presented the highest number of introns selected from BLAST analysis (531 introns) and a high number of predicted mirtrons: 130 by cnnMirtronPred, 150 by pre-miRNAClassification, and 123 classified with high confidence. In contrast, *M. musculus* showed fewer predictions, with only 2 mirtrons confirmed by both tools from a smaller candidate set (74 introns).

**Table 2:** Results of each step in the *in silico* pipeline for the prediction of potential novel mirtrons.^a^

Species	BLAST	cnnMirt	miRNAClass	High	Moderate
*H. sapiens*	242	6	13	4	2
*B. taurus*	4	–	–	–	–
*C. familiaris*	3	–	–	–	–
*M. mulatta*	78	18	22	18	2
*M. musculus*	74	2	3	2	1
*P. troglodytes*	531	130	150	123	13

a BLAST shows introns filtered by sequence similarity; cnnMir and miRNAClass report predictions obtained independently by each classifier, cnnMirtronPred and pre-miRNAClassification, respectively; and the High and Moderate columns represent the confidence levels of the final predictions, indicating the number of mirtrons classified with high or moderate confidence.

For *H. sapiens* and *M. musculus*, the predicted introns showed sequence similarity exclusively with mirtrons annotated from their respective species. In contrast, for *M. mulatta* and *P. troglodytes*, sequence similarity across species was a key determinant factor. Specifically, in *M. mulatta*, among the 18 mirtrons predicted with high confidence, only two showed similarity with known *M. mulatta* mirtrons, while 14 were identified based on similarity to *H. sapiens* mirtrons, and 2 showed similarity to both *H. sapiens* and *P. troglodytes*. For *P. troglodytes*, considering the 123 mirtrons predicted with high confidence, 122 were identified through similarity with *H. sapiens* mirtrons, highlighting the strong conservation of mirtron sequences between these two species.

For *C. familiaris* and *B. taurus*, only 3 and 4 introns, respectively, were retrieved from the BLAST step. However, none of these sequences passed the length filter based on the maximum precursor size threshold, and therefore they were not included in the classification analysis using the prediction tools.

### 3.3 Mirtron availability in miRNA databases

Our findings reveal a relatively low degree of overlap between mirtronDB 2.0 and the reference databases. Specifically, only 950 (38.5%) of mature mirtrons were found only in miRBase, 3 (0.1%) only in MirGeneDB, and 11 (0.4%) mature mirtrons were found in both databases (Table 3). These results show that a substantial portion of the mature mirtrons in mirtronDB are not covered by widely referenced miRNA databases, highlighting the originality and importance of the data offered by mirtronDB.

**Table 3:** Mature mirtrons availability in miRBase, and MirGeneDB, and data exclusively presented in mirtronDB.

	miRBase v22.1		MirGeneDB v3.0		miRBase and MirGeneDB		mirtronDB	Exclusively available in mirtronDB	
Organism Group	#	%	#	%	#	%	#	#	%
*H. sapiens*/*M. musculus*	867	39.9	2	0.1	0	0.0	2173	1304	60
Other chordates	24	41.4	1	1.7	0	0.0	58	33	56.9
Invertebrate	56	71.8	0	0.0	11	14.1	78	11	14.1
Plant	3	2.2	0	0.0	0	0.0	139	136	97.8
Protist	0	0.0	0	0.0	0	0.0	22	22	100
Total	950	38.5	3	0.1	11	0.4	2470	1506	61.0

## 4 Implementation, web portal, and statistics

### 4.1 Website and download

The mirtronDB web portal offers user-friendly access to comprehensive data, built on PostgreSQL 11.7 (Debian 11.7–0+deb10u1), PHP 7.3.19–1, and JavaScript ES6. All information is organized by species and available in multiple file formats. Users can download data as detailed CSV files (including all relevant information), GFF annotation files, BED files, and FASTA sequence files. Downloads can be customized to include precursor sequences, mature sequences, or both. All the data for versions 1.0 and 2.0 are available in the Figshare repository (see the Data availability section).

### 4.2 Dashboard and network

We have also upgraded the mirtronDB web portal to include graphical visualizations of the database. mirtronDB now features three interactive pie charts displaying the number of mature and precursor mirtrons by species, as well as the number of articles included in our dataset, grouped by year of publication. Additionally, a phylogenetic tree is presented, organized by species groups (Chordates, Plants, and Invertebrates). Finally, two interactive box plots are available, showing the sequence length and GC content of the mature mirtrons. For the network, we ran the miRanda tool, and the output totaled >10 million interactions (10 114 676 results). To avoid false positives, we filter and consider only the top 50 best-score interactions for each mature mitron (final total: 56 950 interactions), which are available on the mirtronDB website and in Table S3, available as supplementary data at *Bioinformatics* online.

## 5 Discussion and conclusion

The mirtronDB 2.0 provides an expanded and curated resource dedicated to mirtrons, incorporating over 8 years of newly available data. Our systematic curation of the recent literature has included 44 new mature sequences, 124 new precursors, and 15 new species. This expansion provides a more comprehensive overview of the mirtron landscape and facilitates future comparative studies on their evolution and regulatory roles across a wider range of organisms.

A key finding from this update is that mirtronDB 2.0 significantly expands the mirtron landscape, highlighting the originality of our database. Our findings demonstrate that 61% of our curated content is not present in widely used miRNA repositories such as miRBase (Kozomara *et al.* 2019) or MirGeneDB (Clarke *et al.* 2025). This limited overlap highlights the importance of maintaining a specialized resource for mirtrons up to date, as general miRNA databases may not capture these non-canonical entries due to differences in their biogenesis pathways (Rorbach *et al.* 2018). Therefore, mirtronDB 2.0 provides a more complete and accurate view of the mirtron landscape.

To further develop the catalog of mirtrons beyond literature-curated entries, we implemented a computational prediction pipeline for six mammalian species. The combined use of BLAST-based similarity searches and length thresholds may be inherently more restrictive for non-model species, particularly when compared to humans, reflecting methodological bias rather than true biological absence. This approach, based on established mirtron criteria, identified 165 new potential mirtrons that still require experimental validation. The integration of these computationally predicted candidates together with experimentally validated mirtrons provides researchers with a useful set of targets for functional studies, accelerating the discovery of new mirtrons (Gao *et al.* 2025). This combined strategy ensures that mirtronDB remains a key resource for mirtron research by offering a comprehensive and up-to-date dataset.

Beyond content growth, we have also focused on enhancing the user experience and accessibility of mirtronDB 2.0. The new website, featuring an interactive dashboard and improved search functionality, allows users to explore the database with greater efficiency. These usability improvements ensure that researchers can quickly access the most relevant data for their studies, reinforcing mirtronDB’s role as a user-friendly tool in the field.

mirtronDB 2.0 represents an updated version to help advance our understanding of mirtrons by providing the most comprehensive and standardized resource to date. By combining literature curation with novel *in silico* predictions, we have expanded the known mirtron landscape and highlighted a critical gap in existing miRNA repositories. The new version of the database, with its enriched content and usability, will serve as a reference for researchers studying mirtron biogenesis, function, and evolution. By providing a more comprehensive and standardized resource, mirtronDB 2.0 is expected to catalyze new discoveries, support comparative analyses, and ultimately contribute to a deeper understanding of non-canonical miRNA biology. Moreover, the growing amount of data on mirtrons within this database can positively impact the development of machine learning-based computational methods, enabling more accurate predictions and deeper biological insights.

## Supplementary Material

btag114_Supplementary_Data

## Data Availability

The mirtronDB webserver is available at http://mirtrondb.cp.utfpr.edu.br/. The complete content of Database 1.0 is freely available in the FigShare repository: https://figshare.com/s/63316eb72fcd8ef31cf1? file=14062844. The complete content of Database 2.0 and the source code for the analyses are also freely available in the FigShare repository: https://figshare.com/articles/dataset/MirtronDB_version2/29344775 with DOI: https://doi.org/10.6084/m9.figshare.29344775. Requests for additional information or resources should be directed to the lead contact, Alexandre R. Paschoal (paschoal@utfpr.edu.br).
