# Supplementary material for: mirtronDB 2.0: enhanced database with novel mirtron discoveries
Source: Bioinformatics. 2026 Mar 11;42(4):btag114. doi: 10.1093/bioinformatics/btag114 (PMC13110858; doi:10.1093/bioinformatics/btag114)
Supplement: btag114_Supplementary_Data [file btag114_supplementary_data.zip › Supplementary_Material.docx]

**MirtronDB 2.0**

**Supplementary Material**

**Contents**

[Supplementary Figure S1. Workflow for literature collection and manual curation of novel mirtrons in mirtronDB 2.0.](#_kca44wgmindb)

[Supplementary Figure S2. Workflow for updating genomic coordinates in mirtronDB 2.0.](#_5fdnbxr32rnw)

[Supplementary Document S1. MirtronDB updates and corrections](#_x8kr621b56z0)

#

# **Supplementary Figure S1.** Workflow for literature collection and manual curation of novel mirtrons in mirtronDB 2.0. A systematic literature search was conducted in PubMed using the keywords “mirtron” AND “mirtrons”, covering publications from 2017 to 2025, and following the same inclusion criteria as mirtronDB 1.0. Articles were initially screened based on language (English only) and the availability of essential information, including species, genomic coordinates, and mirtron sequences. Studies lacking these data were excluded from further analysis. For each eligible article, all mirtron-related information was manually extracted and compiled into a file (.csv). All data, when available, such as species, genomic location, sequence, host gene, experimental context, and reference information, were manually extracted and used as input for the database. Next, we imported using direct SQL commands to populate the “Mirtron Details” fields of the database (e.g., <http://mirtrondb.cp.utfpr.edu.br/fetch_details.php?mrt_details=bta-mirtron-1014>). To ensure non-redundancy, all newly curated entries were systematically compared against mirtronDB 1.0 using scripts that compared species, genomic coordinates, and sequence information. Entries matching previously reported mirtrons were removed, and only novel, non-duplicated mirtrons were retained. Finally, the validated metadata were imported into the mirtronDB 2.0 SQL database using a dedicated data-ingestion script, ensuring consistency between the curated metadata and the database schema.


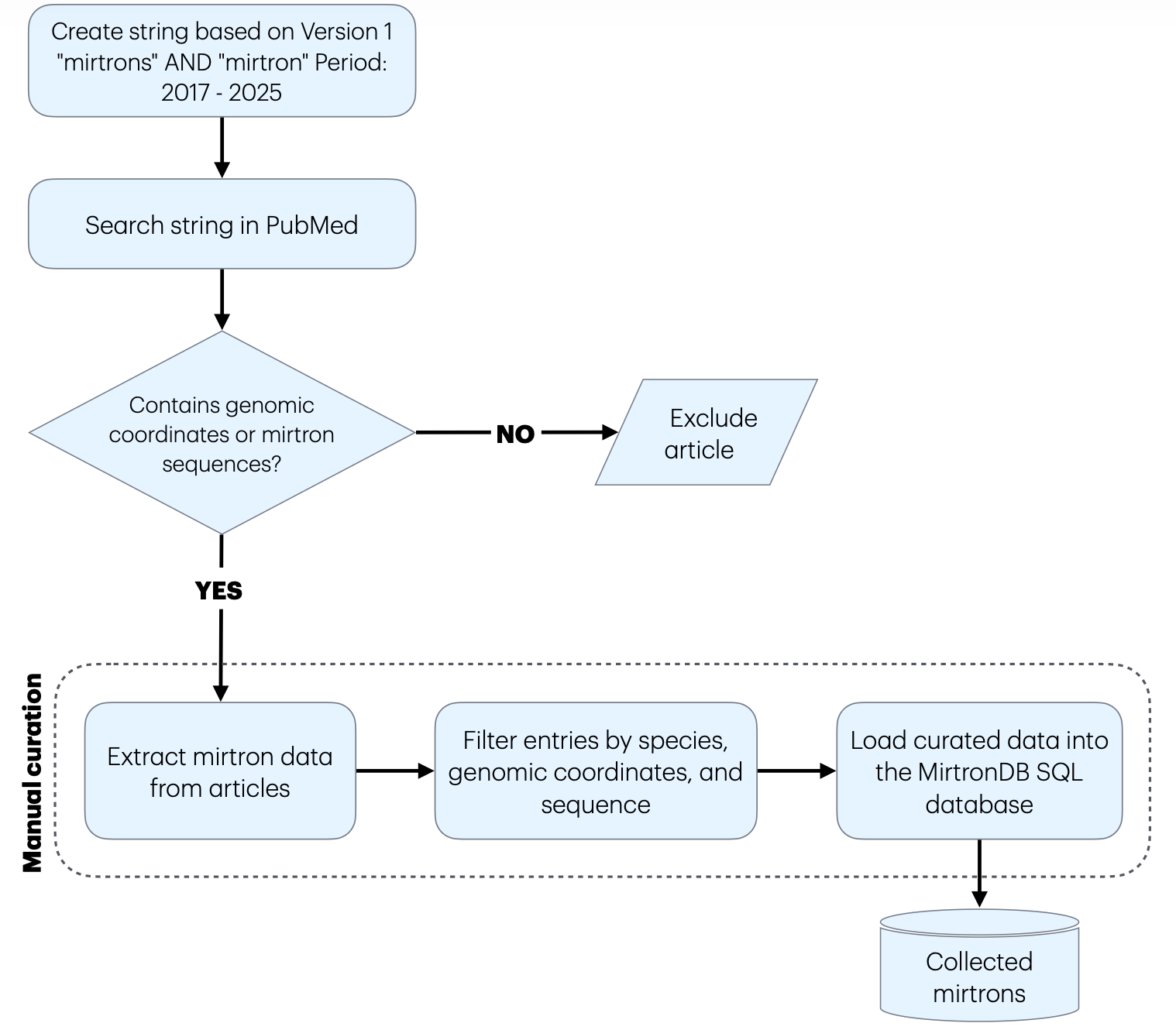


# **Supplementary Figure S2.** Workflow for updating genomic coordinates in mirtronDB 2.0.

**
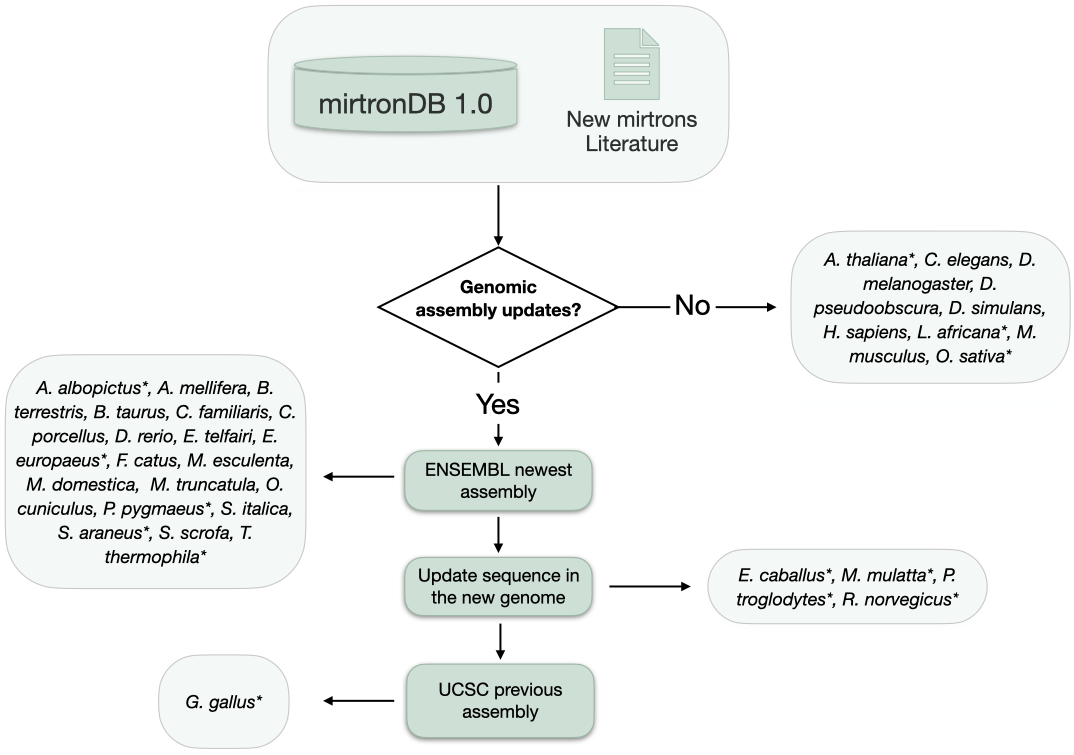
**

All mirtrons from the 33 species included in mirtronDB 2.0 were evaluated to determine whether their genome assemblies matched the latest versions available in the Ensembl database. For species with outdated assemblies, genomic coordinates were updated according to the most recent Ensembl release. If a mirtron sequence exhibited 100% identity in the previous genome assembly and at least 90% similarity in the updated assembly, the sequence was updated accordingly. If the similarity to the newest assembly was below 90%, the mirtron was instead aligned to the penultimate genome assembly available. Species processed at each step of the workflow are indicated within the respective step. Additional details for species marked with an asterisk (*) are provided in Supplementary Text S1.

# **Supplementary Document S1.** MirtronDB updates and corrections

***Aedes albopictus***

aal-mirtron-11893

The mirtrons aal-mirtron-11893-1 and aal-mirtron-11893-2 are annotated as paralogs in the original study. However, when mapped to the current *Aedes albopictus* genome available in Ensembl, the sequence aligns only in one genomic region. Therefore, in mirtronDB, only the version that matches the most recent Ensembl annotation will be retained, following the current genome assembly.

aal-mirtron-11900-1

The mirtrons aal-mirtron-11900-1, aal-mirtron-11900-2, and aal-mirtron-11900-3 are annotated as paralogs in the original study. However, when mapped to the current *Aedes albopictus* genome available in Ensembl, the sequence aligns only in two genomic region. Therefore, in mirtronDB, only the versions that match the most recent Ensembl annotation will be retained, following the current genome assembly.

***Arabidopsis thaliana***

ath-mirtron-1012

It maps two regions. The original article only shows sequence, not coordinates. We mapped the paralogs ath-mirtron-1012-1 and ath-mirtron-1012-2.

***Erinaceus europaeus***

ehd-mirtron-1224

We update it using UCSC Genome Browser instead of Ensembl.

***Equus caballus***

eca-mirtron-nm_017865_6

It’s 96.6% similar to the most updated genome assembly. The sequence was updated in the new genome. We updated the sequence to:

GTAACCTGATGGCTGAGACAGAGGGCAGGGGCATCCTGCAACATGGCTCTCTCTTGAGGCCCTCTGCTTCCTCTTTGCTGCCCACAG

**original sequence from the article:** GTAACCTGATGGCTGAGACAGAGGGCAGGGGCATCCTGCAACATGGCTCTCTCTTGAGGCCCTCTGC**T**TTCCTCTTTGCTGCCCACAG

***Gallus gallus***

As three mirtrons didn’t show good similarity (< 90%) in bGalGal1.mat.broiler.GRCg7b, we opted to update *G. gallus* to GalGal6.

In GalGal6:

gga-mirtron-1201

It’s 98.6% similar to GalGal6 genome assembly. We updated the sequence to:

GTAAGAAACAAACATGATGTGCACGGCTAAGAAAGTGATTTAACTTTTGCTCTGTTTTGCCTGGTTGCCAACCTGGATGC**G**AT**C**A**G**AGCATTGGTAACTAACCTGCAGGCTTACAGACTGCCATAACTTTGCAGAGCTGCACAGTATTTAGTGATTTTGGTGAAGTTTCCAGTGTTCTCCTGTTTTTACCACTGTTTTCTCTTGATATCCCTCCTTAAGCTGTTGGAGGAGACGAGCTTCAAATTTGAGCAGAACCTCAGTCTCTTGCAATGCTTATATTTCAG

**original sequence from the article:**

GTAAGAAACAAACATGATGTGCACGGCTAAGAAAGTGATTTAACTTTTGCTCTGTTTTGCCTGGTTGCCAACCTGGATGC**C**A**T**T**T**AAGCATTGGTAACTAACCTGCAGGCTTACAGACTGCCATAACTTTGCAGAGCTGCACAGTATTTAGTGATTTTGGTGAAGTTTCCAGTGTTCTCCTGTTTTTACCACTGTTTTCTCTTGATATCCCTCCTTAAGCTGTTGGAGGAGACGAGCTTCAAATTTGAGCAGAACCTCAGTCTCTTGCAATGCTTATATTTCAG

gga-mirtron-1202

It’s 98.4% similar to GalGal6 genome assembly. We updated the sequence to:

GTAAGGGCCCTTCCTAAACCTGGGGCGCAGCGACAGTTTGCACTTCGCCTGCTCTGAACCTCCTGGTTAGCAGAGAGGTGACCATGCGTGCAAGCAGGCAGCAGGAGCTGGAGCTCCGGGGGTGCCGGTATGAAAGAAGGGGCTGCAGTGTCCTTCTCCCCCCCCCCCGGCTGTGTTGGCAG

**original sequence from the article:**

GTAAGGGCCCTTCCTAAACCTGGGGCGCAGCGACAGTTTGCACTTCGCCTGCTCTGAACCTCCTGGTTAGCAGAGAGGTGACCATGCGTGCAAGCAGGCAGCAGGAGCTGGAGCTCCGGGGGTGCCGGTATGAAAGAAGGGGCTGCAGTGTCCTTCT**C**CCCCCCCCCCCGGCTGTGTTGGCAG

gga-mirtron-1212

It’s 95.8% similar to GalGal6 genome assembly. We updated the sequence to:

GTGAGCGCGCGGCCCGGTTCGCGGTGCTGCCTCCTCCCTTCCCACCTTAACCGCGTCTCCCTCCCCGCAG

**original sequence from the article:**

GTGAGCGCGCGGCCCGGTTCGCGGTGCTGCCTCCT**C**CCCTTCCCACCTTAACCGCGTCTCCCTCCCCGCAG

gga-mirtron-1219

It’s 90.16% similar to GalGal6 genome assembly. We updated the sequence to:

GTTTGTGGGATGA**G**TGATAGGCT**G**TGTGC**G**CT**T**CTG**A**ATTGCCTCTCTTTTTTCCTT**G**AAC

**original sequence from the article:**

GTTTGTGGGATGA**A**TGATAGGCT**A**TGTGC**A**CT**A**CTG**C**ATTGCCTCTCTTTTTTCCTT**T**AAC

***Loxodonta africana***

Two of the four mirtrons didn’t show a good similarity (< 90%) in the new genome (loxAfr3). We opted to keep the original from the article.

***Macaca mulatta***

mml-mirtron-1820

It’s 97.62% similar to the most updated genome assembly. We updated the sequence to:

AGAAGGTGGGCCTGGGTCGGTGGGGACGGGGCGGCTGGGCGTGCCCTGCGGCCGCTGCTCTAACCGC**G**C**T**GTCCCCCAGGCCCT

**original sequence from the article:**

AGAAGGTGGGCCTGGGTCGGTGGGGACGGGGCGGCTGGGCGTGCCCTGCGGCCGCTGCTCTAACCGC**A**C**C**GTCCCCCAGGCCCT

***Oryza sativa***

osa-mirtron-2400

It maps two regions. As the original article only shows sequence, and not coordinates, we mapped the paralog osa-mirtron-2400-1 and osa-mirtron-2400-2.

***Pan troglodytes***

ptr-mirtron-1233-3

It’s 98.8% similar to the most updated genome assembly. The sequence was updated in the new genome. We updated the sequence to:

TGAGTGGGAGG**C**CAGTGCACGGCAGGGGGAGCTGCAGGGCCGTCGGAGGGGCCCCAGCGTCTGAGCCCTGTCCTCCCGCAG

**original sequence from the article:**

UGAGUGGGAGGCAGUGCACGGCAGGGGGAGCUGCAGGGCCGUCGGAGGGGCCCCAGCGUCUGAGCCCUGUCCUCCCGCAG

ptr-mirtron-nm_015232_11

It’s 98.9% similar to the most updated genome assembly. The sequence was updated in the new genome. We updated the sequence to:

GTAAGTGGAGCCCTAGTTGCCCAGGTGAGTGACTACCCTG**T**CCCCTTGTTCTTCTTCTCACCGTCTCTGTTGTTTCTCTTCTCAG

**original sequence from the article:**

GTAAGTGGAGCCCTAGTTGCCCAGGTGAGTGACTACCCTG**C**CCCCTTGTTCTTCTTCTCACCGTCTCTGTTGTTTCTCTTCTCAG

ptr-mirtron-NM_032635_0

We update it using UCSC Genome Browser instead of Ensembl.

***Pongo pygmaeus***

We considered the genome of Pongo abelii (ponAbe2).

***Rattus norvegicus***

rno-novel-mirtron-41

It’s 98.75% similar to the most updated genome assembly. We updated the sequence to:

GTGAGTGGACATACTGTA**C**AAGGTAGAGGTGGGAGCTCTGCCCTTGTCGGTCCTTATGG**C**CTCTCCTATTCTCCATCTAG

**original sequence from the article:**

GTGAGTGGACATACTGTAAAGGTAGAGGTGGGAGCTCTGCCCTTGTCGGTCCTTATGGCTCTCCTATTCTCCATCTAG

***Sorex araneus***

bca-mirtron-nm_032635_0

We update it using UCSC Genome Browser instead of Ensembl.
